# Biomechanical effect of proximal multifidus injury on adjacent segments during posterior lumbar interbody fusion: a finite element study

**DOI:** 10.1186/s12891-023-06649-7

**Published:** 2023-06-24

**Authors:** Wei Wei, Tianhao Wang, Jianheng Liu, Keya Mao, Chun’ang Pan, Hui Li, Yongfei Zhao

**Affiliations:** 1 Medical School of Chinese PLA, Beijing, 100048 China; 2 Department of Orthopaedics, the First Medical Center, Chinese PLA General Hospital, Beijing, 100048 China; 3 Beijing Engineering and Technology Research Center for Medical Endoplants, Beijing, 100082 China; 4 Beijing Engineering Laboratory of Functional Medical Materials and Instruments, Beijing, 100082 China

**Keywords:** Posterior lumbar interbody fusion, Proximal multifidus, Facet joint violation, Adjacent segment degeneration, Finite element analysis, Biomechanics

## Abstract

**Background:**

Adjacent segment degeneration (ASD) is a common complication of lumbar interbody fusion; the paraspinal muscles significantly maintain spinal biomechanical stability. This study aims to investigate the biomechanical effects of proximal multifidus injury on adjacent segments during posterior lumbar interbody fusion (PLIF).

**Methods:**

Data from a lumbosacral vertebral computed tomography scan of a healthy adult male volunteer were used to establish a normal lumbosacral vertebral finite element model and load the muscle force of the multifidus. A normal model, an L4/5 PLIF model (PFM) based on a preserved proximal multifidus, a total laminectomy PLIF model (TLPFM), and a hemi-laminectomy PLIF model based on a severed proximal multifidus were established, respectively. The range of motion (ROM) and maximum von Mises stress of the upper and lower adjacent segments were analyzed along with the total work of the multifidus muscle force.

**Results:**

This model verified that the ROMs of all segments with four degrees of freedom were similar to those obtained in previous research data, which validated the model. PLIF resulted in an increased ROM and maximum von Mises stress in the upper and lower adjacent segments. The ROM and maximum von Mises stress in the TLPFM were most evident in the upper adjacent segment, except for lateral bending. The ROM of the lower adjacent segment increased most significantly in the PFM in flexion and extension and increased most significantly in the TLPFM in lateral bending and axial rotation, whereas the maximum von Mises stress of the lower adjacent segment increased the most in the TLPFM, except in flexion. The muscle force and work of the multifidus were the greatest in the TLPFM.

**Conclusions:**

PLIF increased the ROM and maximum von Mises stress in adjacent cranial segments. The preservation of the proximal multifidus muscle contributes to the maintenance of the physiological mechanical behavior of adjacent segments, thus preventing the occurrence and development of ASD.

## Background

Posterior lumbar interbody fusion (PLIF) is the “gold standard” treatment of degenerative lumbar spondylolisthesis, lumbar disc herniation, and spinal deformity; it has broad indications, a precise decompression effect, and a high fusion rate [1, 2]. Although many innovative surgical approaches rely on minimally invasive techniques or other invasive approaches, the traditional PLIF is irreplaceable [3, 4]. Moreover, adjacent segment degeneration (ASD) is a serious complication facing spinal surgeons, for which, iatrogenic injury to key posterior structures, such as the paraspinal muscles and ligaments, during the posterior approach is a crucial risk factor [5–7].

Although the specific mechanism of ASD remains unclear, pathological changes in the kinematics and dynamics of adjacent segments are widely recognized as important mechanical mechanisms for its development after lumbar interbody fusion [8]. An obstruction in the skin, adipose tissue, or paraspinal muscle during surgery may affect the entry point or direction of the pedicle screw, resulting in a disturbance or injury to the facet joint (FJ), which when occurs in the upper adjacent segment accelerates the occurrence of ASD [5, 9]. The relationship between the preservation of the proximal FJs and the prevention of ASD development has been widely recognized [10, 11].

According to the anatomical description, the multifidus, which is the most critical muscle for the stability of the lumbar spine, terminates at the mammillary process down across the level and is extremely near to the FJ [12, 13]. Kim et al. compared different posterior approaches to the lumbar spine and found that preserving the tendon initiation in the multifidus and reducing muscle dissection in the spinous process could prevent postoperative multifidus atrophy [14]. During PLIF, the violation of the FJ through pedicle screw placement or the decompression of the narrow spinal canal is likely associated with severing or injury of the multifidus muscle bundle. The atrophy and disuse of the multifidus due to extensive dissection and severing, which decrease muscle strength and spinal alignment instability, may accelerate the occurrence of ASD postoperatively and significantly affect postoperative clinical outcomes [15].

Recently, clinical studies have shown that the effective cross-sectional area (eCSA) of the multifidus is related to the occurrence of ASD; however, most studies are limited to measuring the eCSA or fat infiltration of the multifidus in the lower lumbar segment via magnetic resonance imaging [16, 17], with a few reported biomechanical mechanism studies. In this study, a finite element (FE) analysis was used to model the multifidus using a novel method and to investigate and quantify the effect of proximal multifidus injury on the biomechanics of adjacent segments after PLIF. We aim to evaluate the effect of proximal multifidus injury on the occurrence of ASD, provide guidance for the effective protection of the multifidus in clinical surgery, and improve the postoperative efficacy and rapid recovery of patients.

## Methods

### The establishment of the musculoskeletal FE model

A 40-year-old healthy male volunteer was recruited for our study, which excluded severe degenerative diseases, tumors, deformities, or trauma involving the lumbar spine. This study is approved by the Institutional Review Board of the First Medical Center of the General Hospital of the People’s Liberation Army, and a written informed consent was obtained from the computed tomography (CT) volunteer. This study was conducted in accordance with the principles of the Declaration of Helsinki. The volunteer’s CT scans of the lumbosacral vertebrae were obtained (0.5 mm thick). The data were stored in the DICOM format, and a 3D model was reconstructed using Mimics 24.0 (Materialise, Belgium). Bone and soft tissues were reverse reconstructed using Geomagic Studio 2013 (Geomagic, USA), component assembly, and FE modeling pre-processing in Abaqus 2016 (SIMULIAInc, USA), and the solid part of the model was partitioned using C3D8R units. Owing to the complexity of the model, only a small number of C3D4 and C3D6 units were used. T3D2 units were used to simulate the ligament structure. The surface contact element was used to simulate the joint surface, and the friction coefficient of the joint surface was 0.1. An FE analysis was then conducted. The organization and structure of the model were assigned material attributes, as listed in Table 1, and the normal lumbosacral vertebral FE model was established.

**Table 1:** Material properties assignment of the FE model

Organization	Young modulus(MPa)	Poisson ratio	Element Type	cross-sectional area(mm2)	Element Size(mm)	Number of elements
Cancellous bone	200	0.45	C3D4	/	2	341,121
Cortical bone	12,000	0.3	C3D8R	/	2	14,071
Annulus fibrosus	4.2	0.45	C3D8R	/	2	7392
End plate	1000	0.4	C3D8R	/	2	5632
Facet	10	0.4	C3D8R	/	2	1087
Fibers	400	0.3	T3D2	0.00015	/	14,781
nucleus pulposus	1	0.48	C3D8R	/	2	3872
Anterior longitudinal	7.8	0.3	T3D2	15.0	/	25
Capsular ligament	7.5	0.3	T3D2	0.3	/	150
Interspinous ligament	10	0.3	T3D2	8.0	/	36
Intertransverse ligament	10	0.3	T3D2	0.2	/	30
Ligamentum fIavum	15	0.3	T3D2	3.0	/	14
Posterior longitudinal	10	0.3	T3D2	2.0	/	25
Supraspinous ligament	10	0.3	T3D2	13.0	/	5
Posterior bone	3500	0.25	C3D4	/	/	148,693

**Fig. 1:**
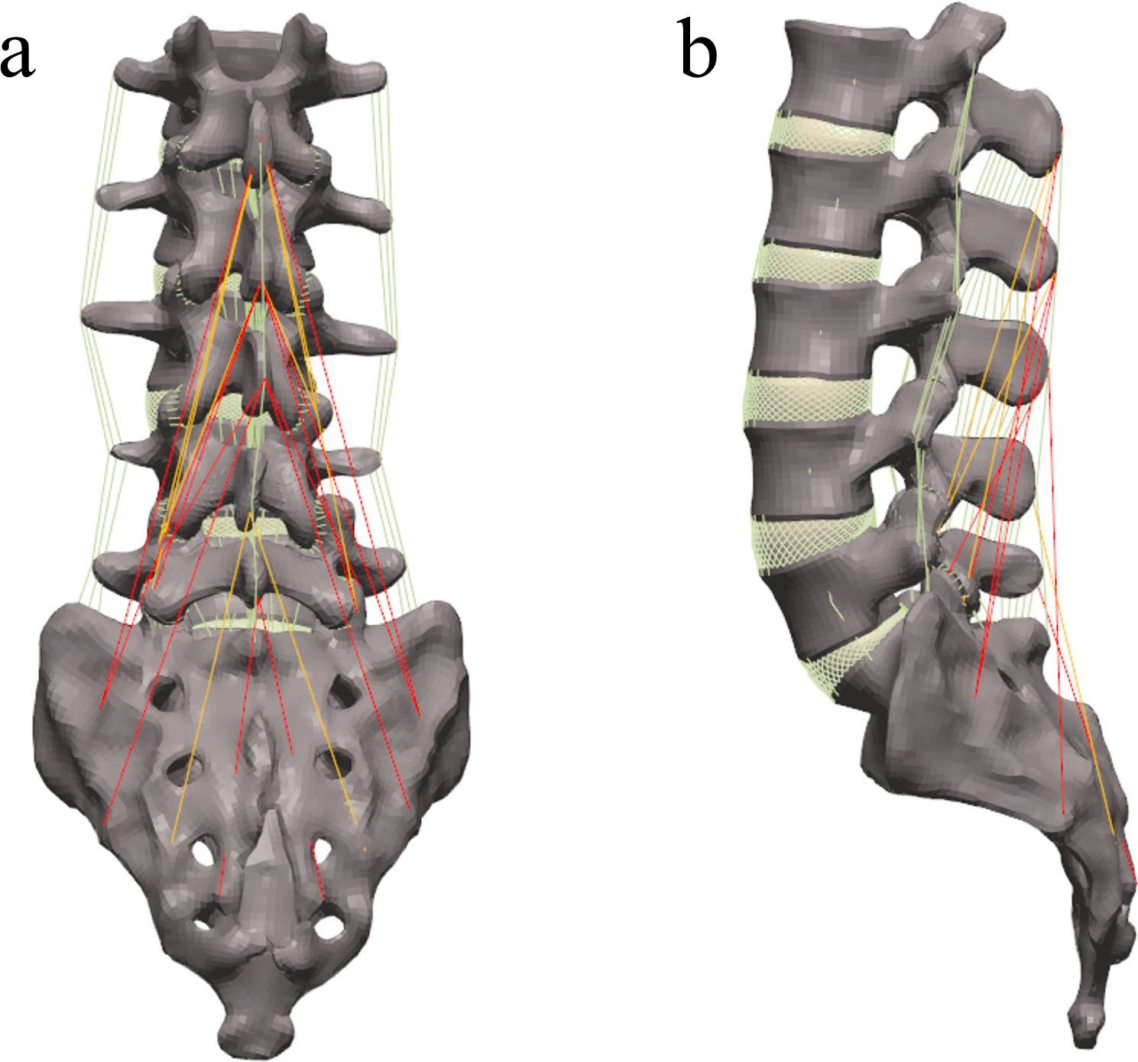
The FE model of the intact lumbosacral spine including the multifidus (**a**) posterior view; (**b**) lateral view (Yellow lines represent the excised proximal multifidus)

In this study, a proven FE model of the lumbosacral vertebrae loaded with a multifidus was used. Based on the anatomical characteristics of the multifidus, reference points were established between the spinous processes and the corresponding mammillary processes at the back of each lumbar vertebrae and were bound using the T3D2 spring units; a total of 11 muscle bundles were established, as shown in Fig. 1. According to the Hill’s study on the skeletal muscle mechanics [18], the muscle tensioning force T0 strongly depends on the muscle retention length L0. The muscle strength of 11 pairs of multifidus muscles followed a fixed proportional relationship, and the muscle force of the multifidus was assigned; thus, this study assumed that muscle force is positively correlated with muscle length. The active tensioning force of the multifidus was adjusted according to the displacement distance and muscle length changes in different motion modes [19].

### Establishment of a PLIF model based on proximal multifidus injury

In this study, a complete physiological model loaded with a multifidus was used as the normal model (NM). L4/5 PLIF was performed. The L4/5 disc was replaced with cancellous bone, and the contact mode with the upper and lower endplates was adjusted to no relative movement. The unloaded spring element model simulated the severing of the multifidus. According to the standard PLIF procedure, one pair of the L4-S multifidus was severed by resecting the bottom half of the L4 spinous process, and the posterior fusion model (PFM) of the L4/5 was obtained.

Similarly, three pairs of the proximal multifidus (L1-L4, L1-L5, and L2-L5) terminating at the L4 and L5 mammillary processes were severed, and L4 total laminectomy and interbody fusion were performed to obtain a PFM of total laminectomy (TLPFM). In addition, the right hemi-laminectomy PFM (HLPFM) protecting the partial proximal multifidus was established, with only two severed multifidus muscles terminating at the left mammillary process of the L5 (L1-L5 and L2-L5 of the left).

### Boundary and loading conditions

In this study, the boundary and loading conditions were based on Yamamoto’s in vitro studies [20]. The motion of the sacrum in all directions was assumed to be limited, and a vertical compressive preload of 500 N was applied to the upper endplate of the L1 vertebra to simulate body weight.

A 10-Nm moment was imposed on the upper endplate of the L1 vertebra, and four directions of motion were completed, including flexion (FL), extension (EX), lateral bending (LB), and axial rotation (AR). Except for the HLPFM, the other three models only used the left LB and left AR instead of the bilateral motions to simplify the experimental procedure. According to Bojairami [19], after adjusting the multifidus tensioning force of the NM, the other surgical groups were set to the connector state.

### Mesh sensitivity analysis

In this study, a mesh sensitivity analysis of the NM was performed. Most of the elements were divided into 8-node hexahedral elements, and the mesh size was changed (3, 2.5, 2.0, 1.5, and 1.0 mm) respectively. Under the same boundary and loading conditions as described above, the movement of the sacral vertebrae in all directions was restricted; a vertical compressive load of 500 N was applied to the upper endplate on the L1, and the maximum intervertebral von Mises stress trend of the L4/5 intervertebral disc was obtained. When the mesh size was of < 1.0 mm, the resulting stress tended to be stable (change of < 5%), and the mesh could be considered convergent [21]. The mesh size was adjusted to 1.0 mm to improve the accuracy of the unit simulation and the convenience of calculation.

### Model validation

**Fig. 2:**
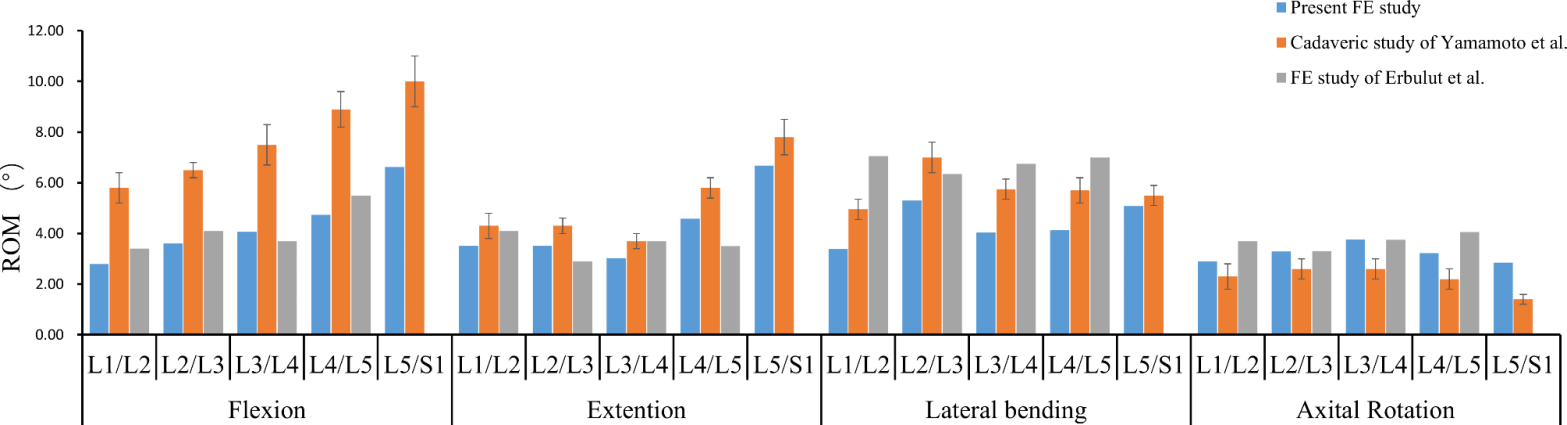
L1-S1 segmental ROM validation results of the intact FE model

A vertical compressive preload of 500 N was applied to the upper endplate of the L1 vertebra of the FE model of the lumbosacral vertebrae without multifidus muscle loading, and a 10-N-m moment was then applied to the FL, EX, LB, and AR motions. Consecutively, the range of motions (ROMs) of each segment was recorded and compared with those obtained from previous biomechanical studies [20, 22]. The comparison results, which are close to the trend of change, are shown in Fig. 2. This proves that this model is effective and can be used in further research.

## Results

### ROM of adjacent segments

**Fig. 3:**
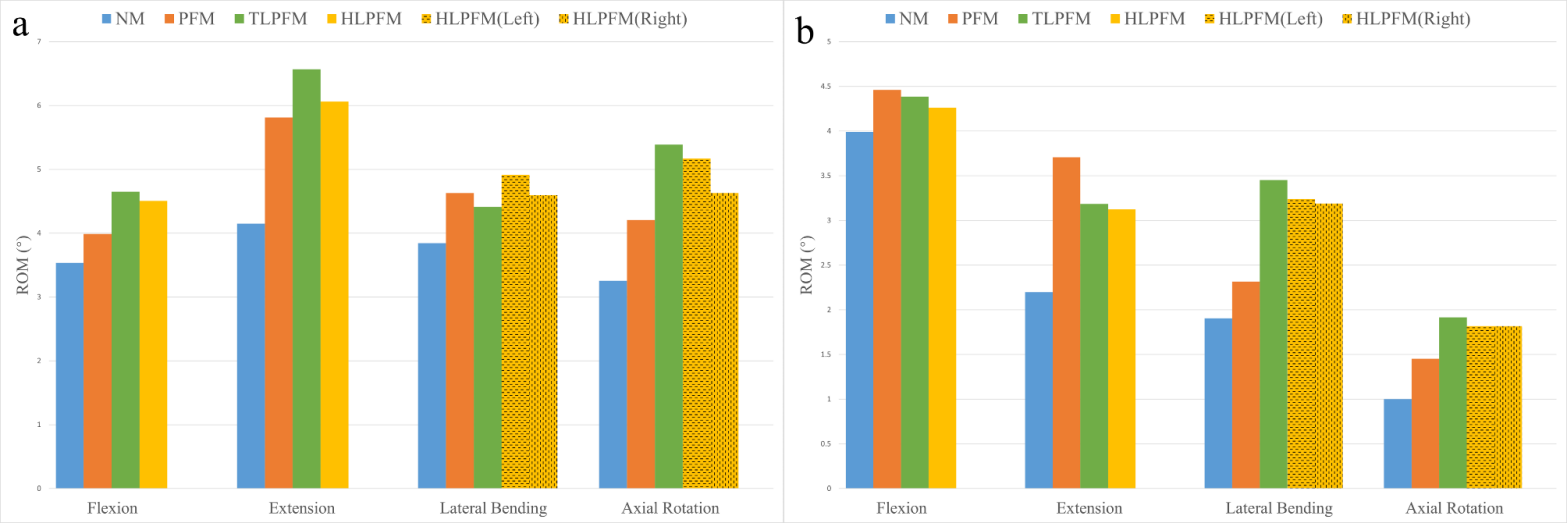
A comparison between the adjacent segmental ROMs (**a**) L3/4; (**b**) L5/S1

Regardless of whether the proximal multifidus muscle was severed, the ROM in the upper and lower adjacent segments increased significantly after L4/5 PLIF was performed. Compared with the ROMs in the NM, the ROMs of the L3/4 in the three other models increased by 12.85–31.53% in FL, 40.06–58.24% in EX, 14.72–27.87% in LB, and 29.24–65.58% in AR. The largest increase was observed in the TLPFM. The ROMs of the L5/S1 increased by 6.75–11.73% in FL, 42.26–68.78% in EX, 21.64–81.42% in LB, and 45.15-91.44% in AR. The PFM showed the largest increase in FL and EX. The TLPFM showed the most increase in LB and AR. Figure 3 compares the ROMs of the adjacent segments with six degrees of freedom for the four models.

### Maximum von Mises stress of adjacent discs

**Fig. 4:**
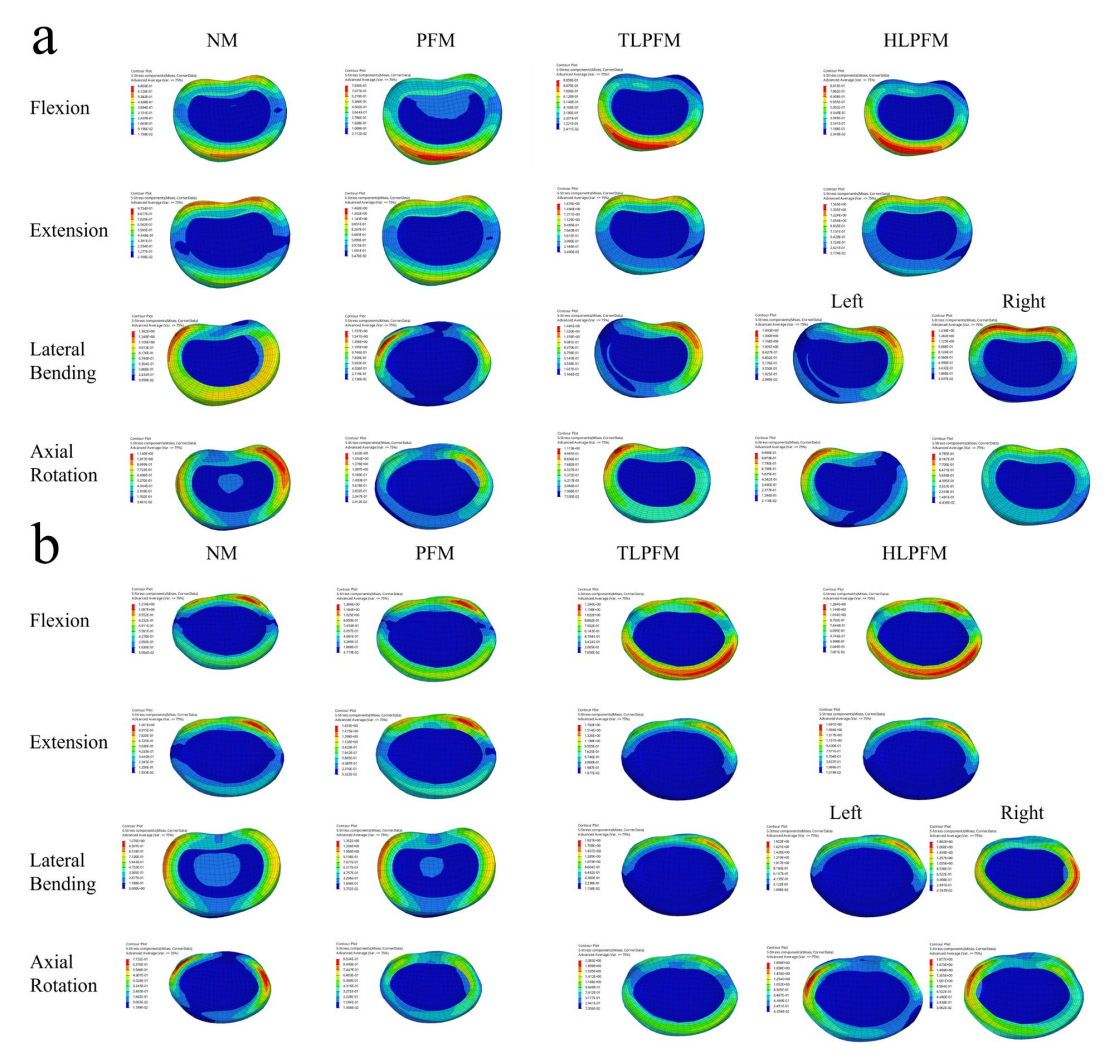
The stress nephogram of the adjacent discs (**a**) L3/4; (**b**) L5/S1

**Fig. 5:**
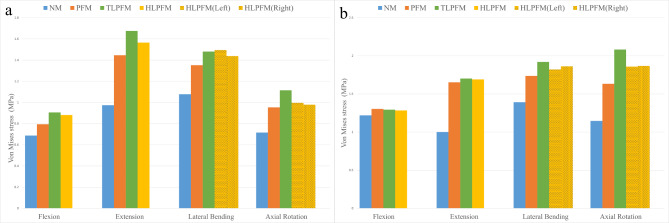
The maximum von Mises stress in adjacent segments (**a**) L3/4; (**b**) L5/S1

The stress nephogram of the upper and lower adjacent discs is shown in Fig. 4; the stress distribution is significantly correlated with the direction of motion. The TLPFM and HLPFM of the severed proximal multifidus muscle showed more obvious stress concentration areas. The stress peaks of the upper and lower adjacent discs increased under all conditions after L4/5 PLIF was performed. The maximum von Mises stress in the L3/4 of the TLPFM in FL and EX increased by 31.88% and 72.19%, respectively, compared with that in the NM. Regarding LB, the largest increase (38.77%) was observed in the left LB in the HLPFM compared with that in the NM. Moreover, the maximum von Mises stress in AR (a 55.90% increase) was detected in the TLPFM compared with that in the NM. Except for FL, the maximum von Mises stress in the L5/S1 showed the most significant increase in the TLPFM (70.03%, 37.98%, and 81.76% in EX, LB, and AR) compared with that in the NM. A comparison of the maximum von Mises stress in the upper and lower adjacent discs is shown in Fig. 5.

### Total work of the multifidus

**Fig. 6:**
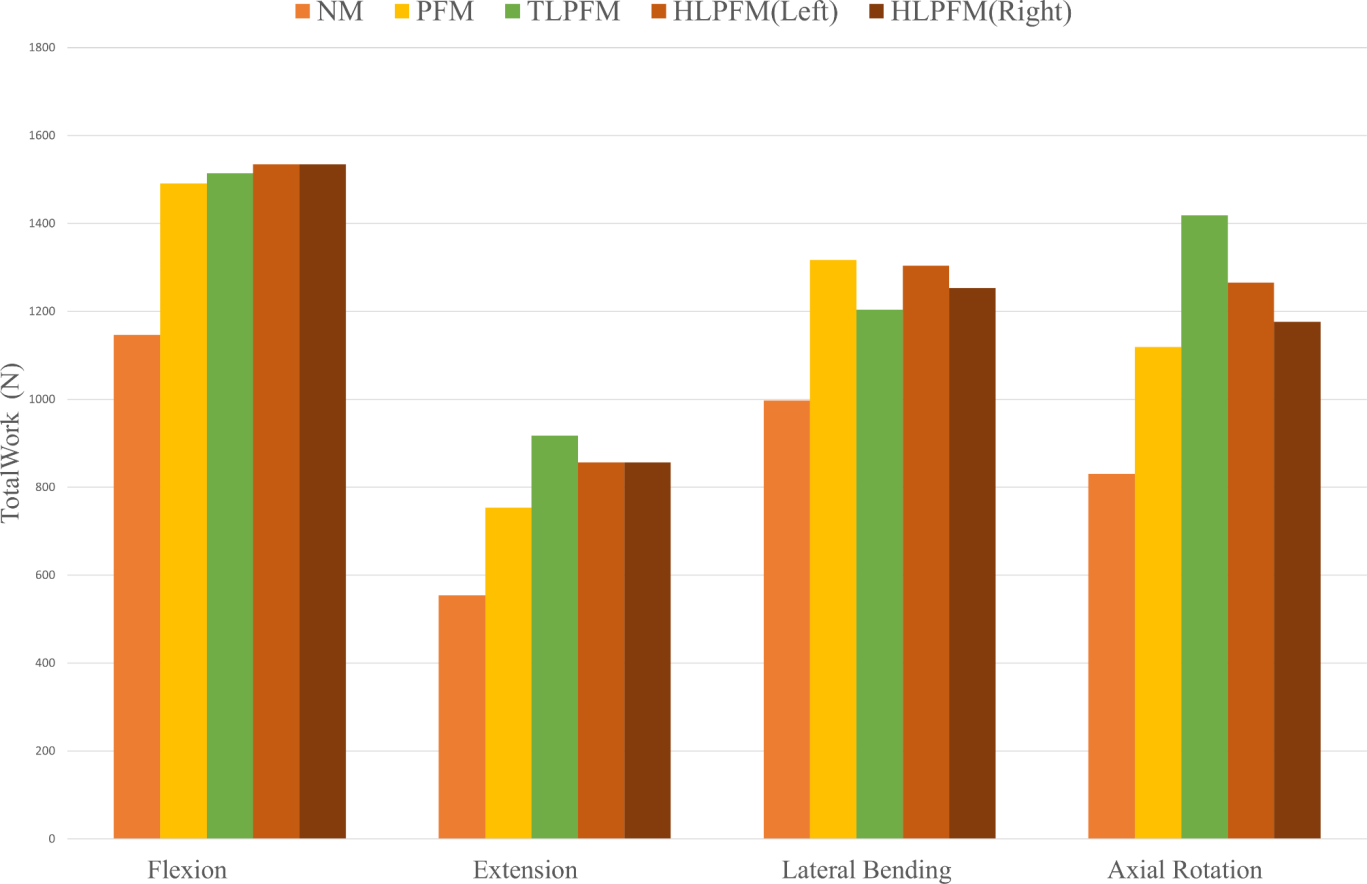
A comparison of total work capacity

The total work capacity by the multifidus muscles in the four models was 3528.32 mJ, 4681.19 mJ, 5054.33 mJ, and 4890.64 mJ, respectively. The work capacity under different motion conditions is shown in Fig. 6.

## Discussion

ASD has become a recognized intermediate and long-term complication of PLIF, necessitating revision surgery and increasing pain and economic burden. Although the mechanism and progression of ASD and its relevance to spinal fusion are still controversial [6, 7], the possible biomechanical influences and risk factors for ASD are well understood. The occurrence of ASD is associated with individual differences, such as sex and body mass index [23], and clearly correlates with the extent of injury, fusion segment selection, and length of fusion [24]. Previous biomechanical and clinical studies have shown that the development of ASD is usually accompanied with abnormal changes in biomechanical factors such as the maximum von Mises stress of the intervertebral annulus fibrosus and the ROM in adjacent segments [8]. The damage to the trunk muscles and ligaments due to PLIF has gained the attention of spine surgeons [15, 26–27], as it is closely related to postoperative rehabilitation and symptom relief. However, anatomically, in addition to obvious intraoperative paraspinal muscle dissection and compression injury [15, 28], the attachment relationship between the paraspinal muscle and bone structure behind the lumbar spine is easier to overlook. It is noteworthy that the deep multifidus is closely related to the superior articular process. In this study, an FE model of the lumbosacral vertebrae loaded with the multifidus was established to investigate and quantify the biomechanical effects of proximal multifidus injury on the adjacent segment after PLIF.

Although this model does not perfectly interpret the complex lumbo-dorsal situation, it can effectively expand the understanding of trunk muscles for the biomechanical stability of adjacent segments after PLIF. In the three models, in which PLIF was performed, the ROM and maximum von Mises stress increased significantly in the upper and lower adjacent segments. This finding was consistent with the previous understanding of the biomechanical behavior of ASD. When fusion occurs, the ROM of the fusion segment is lost, and stress concentration leads to the diffusion of biomechanical properties to adjacent segments [26]. After the proximal multifidus muscle was severed, the ROM in the upper adjacent segment increased by 27.48–58.24% in FL and EX, and the maximum von Mises stress increased by 28.33–72.19%, which both showed more significant instability than that in the PFM. The changes in the ROM in the lower adjacent segment in FL and EX were partially similar to those in the upper adjacent segment, and the maximum von Mises stress of the adjacent segments reflected more stress conduction effects after fusion. Moreover, most biomechanical activities of the adjacent segments can be effectively protected by preserving the proximal multifidus muscle.

The multifidus, the most important trunk muscle to provide lumbar stability [29], provides approximately 2/3 of the stability force in the lumbosacral region [30], while the lumbar muscle has a wide ROM and a high frequency of activity; therefore, the lumbosacral multifidus is the most prone to strain. Duan et al. [17] conducted a retrospective study on 178 patients with lumbar spondylolisthesis and graded the fat infiltration of the lumbar multifidus muscle using magnetic resonance imaging. They found that higher postoperative fat infiltration might be associated with ASD as the loss of effective muscle cross-sectional area (decreased muscle mass) caused by fat infiltration or muscle atrophy is an important risk factor for the accelerated development of ASD [25, 31, 32]. In addition, the lumbosacral multifidus muscle of patients with chronic low back pain has a higher fatigue rate than that of healthy individuals, whereas no significant difference in other trunk muscles, such as the iliocostalis lumborum muscle, is observed [33]. Our study, which compared the work capacity of the multifidus muscle, inferred that the increase in work capacity caused by severing the multifidus muscle was the most significant. For an upright neutral position, the overactivation of the trunk muscles has the opposite effect on the stability of the spine [34], and the active role of the multifidus muscle is more reflected in preventing excessive forward FL and slip of the spine; imbalances caused by muscle force can lead to more severe instability [29].

Previous studies stated that during PLIF surgery, the FJ would be invaded during proximal segment pedicle screw placement, thus accelerating the development of ASD [11, 36–37]. The cranial FJ belongs to the upper adjacent segment that forms the functional spinal unit, and an injury to its lower segment upsets the local balance, thus endangering the upper adjacent segment. Cortical bone trajectory (CBT) pedicle screws produce smaller FJ infestations and paraspinal muscle injuries owing to their greater caudomedial starting point. Sakaura et al. [4] compared CBT pedicle screws with traditional trajectory pedicle screws for single-level degenerative lumbar disease and found that the CBT group had a lower incidence of postoperative ASD. The current mainstream view focuses more on maintaining the integrity of the FJ, which is explained by the structural integrity of the posterior column [26]. We established HLPFM based on the fact that in an actual clinical surgery, even if only one side of the FJ and its corresponding affiliated muscles were preserved during the decompression of hemi-laminectomy, relatively more physiological mechanical activity attributes could be retained.

Based on anatomical and imaging studies of cadaveric specimens and volunteers [12, 13], all the bundles of the multifidus muscle are closely clustered, fanned out from the posterior margin of the spinous process downwards, spanning 1–2 segments, and attached to the corresponding vertebral mamillary process. Attachment points were located no more than 3 mm from the lateral side of the superior articular process, thus, forming a very close relationship with the FJ rendering it susceptible to being severed or injured inadvertently. The posterior formation of multiple “bowstring effects” of the multifidus muscle is extremely important for the physiological lordosis of the lumbar spine [38]. Liu et al. [39] found that multifidus atrophy was associated with a high incidence of lumbar disc herniation. Although the particular mechanical mechanism is unknown, it is critical in preserving the multifidus muscle during surgery. Aono et al. [3] performed L3/4 PLIF for 71 patients and found that ASD occurred more frequently at the L4/5 caudally, which they hypothesized was due to a higher stress concentration and motion at the L4/5 postoperatively, as well as a higher incidence of natural degeneration at the L4/5. However, according to the results of the current study, TLPFM severed two pairs of the multifidus muscles ending at the L5 FJ, and compared with the PFM, its upper adjacent segments showed similar changes, and its lower adjacent segments showed significant changes in the ROM and maximum von Mises stress in LB and AR, respectively. The unexpected change in the biomechanical behavior of the lower adjacent segments caused by the severing of the multifidus muscle, which begins and ends at the cranial side of the fusion segment, cannot be explained by the positive effect of single multifidus muscle bundle retention. Thus, we speculate that the multi-muscle bundle synergistic effect of the multifidus plays an important role in maintaining the stability of the lumbar spine sequence during coronal and cross-sectional movements.

However, this study had some limitations. First, we subjectively speculated that the muscle completely lost its ability to actively contract after being severed without taking into account the scar healing and muscle reconstruction that occur in real setting. Second, regarding the complex and changeable human spinal movements, only the mechanical parameter changes in the FE analysis were used to predict the actual postoperative rehabilitation process, which might have caused an obvious bias. However, this study considered the iatrogenic injury of the muscle as the starting point to analyze the specific situation that might occur postoperatively and obtained important preliminary results, which are positively significant for further spinal musculoskeletal research. Therefore, further investigations can be performed using this method.

## Conclusions

The PLIF models showed significant abnormal increases in the ROM and maximum von Mises stress, and the changes in the upper adjacent segments were more significant than those in the lower adjacent segments. The model with the preserved proximal multifidus muscle retained a relatively more physiological mechanical behavior of the adjacent segment, and hemi-laminectomy had some protective significance for the adjacent segment. Thus, preserving the integrity of the proximal multifidus muscle prevents the occurrence and development of ASD.

## Data Availability

The datasets used and/or analyzed during the current study are available from the corresponding author on reasonable request.

## List of Abbreviations

ASD: Adjacent segment degeneration
PLIF: Posterior lumbar interbody fusion
PFM: L4/5 PLIF model
TLPFM: Total laminectomy PLIF model
HLPFM: Hemi-laminectomy PLIF model
ROM: Range of motion
FJ: Facet joint
eCSA: Effective cross-sectional area
FE: Finite element
CT: Computed tomography
NM: Normal model
FL: Flexion
EX: Extension
LB: Lateral bending
AR: Axial rotation
CBT: Cortical bone trajectory
